# Predictors of transition in patients with clinical high risk for psychosis: an umbrella review

**DOI:** 10.1038/s41398-023-02586-0

**Published:** 2023-08-28

**Authors:** Christina Andreou, Sofia Eickhoff, Marco Heide, Renate de Bock, Jonas Obleser, Stefan Borgwardt

**Affiliations:** 1 Translational Psychiatry, Department of Psychiatry and Psychotherapy, University of Lübeck, Ratzeburger Allee 160, 23562 Lübeck, Germany; 2 Center of Brain, Behavior, and Metabolism (CBBM), University of Lübeck, Ratzeburger Allee 160, 23562 Lübeck, Germany; 3 University Psychiatric Clinics Basel, Wilhelm Klein-Strasse 27, 4002 Basel, Switzerland; 4 Department of Psychology, University of Lübeck, Ratzeburger Allee 160, 23562 Lübeck, Germany

**Keywords:** Prognostic markers, Schizophrenia

## Abstract

Diagnosis of a clinical high-risk (CHR) state enables timely treatment of individuals at risk for a psychotic disorder, thereby contributing to improving illness outcomes. However, only a minority of patients diagnosed with CHR will make the transition to overt psychosis. To identify patients most likely to benefit from early intervention, several studies have investigated characteristics that distinguish CHR patients who will later develop a psychotic disorder from those who will not. We aimed to summarize evidence from systematic reviews and meta-analyses on predictors of transition to psychosis in CHR patients, among characteristics and biomarkers assessed at baseline. A systematic search was conducted in Pubmed, Scopus, PsychInfo and Cochrane databases to identify reviews and meta-analyses of studies that investigated specific baseline predictors or biomarkers for transition to psychosis in CHR patients using a cross-sectional or longitudinal design. Non-peer-reviewed publications, gray literature, narrative reviews and publications not written in English were excluded from analyses. We provide a narrative synthesis of results from all included reviews and meta-analyses. For each included publication, we indicate the number of studies cited in each domain and its quality rating. A total of 40 publications (21 systematic reviews and 19 meta-analyses) that reviewed a total of 272 original studies qualified for inclusion. Baseline predictors most consistently associated with later transition included clinical characteristics such as attenuated psychotic and negative symptoms and functioning, verbal memory deficits and the electrophysiological marker of mismatch negativity. Few predictors reached a level of evidence sufficient to inform clinical practice, reflecting generalizability issues in a field characterized by studies with small, heterogeneous samples and relatively few transition events. Sample pooling and harmonization of methods across sites and projects are necessary to overcome these limitations.

## Introduction

The paradigm of indicated prevention for psychotic disorders was introduced in the 1990s based on the observation that the majority of patients with a first psychotic episode reported retrospectively a *prodromal* phase that preceded the onset of overt symptoms by several years and was characterized by subthreshold or unspecific symptoms, and/or functional decline [1]. The new paradigm provided a new and promising perspective for improving the course of these often chronic and severe disorders. The operationalization of diagnostic criteria for of a clinical high-risk state (CHR), also referred to as ‘at-risk mental state’ (ARMS), enabled early detection and timely treatment for individuals at risk before the onset of overt psychotic symptoms, which may contribute to delaying or preventing the first manifestation of a psychotic disorder, and improve their clinical and functional outcomes [2].

Diagnosis of a clinical high-risk state is based on specific sets of clinical criteria. The most widely established of those are ultra-high-risk (UHR) criteria, which require one of the following for a CHR diagnosis [3]: (a) attenuated psychotic symptoms symptoms (APS), i.e., positive symptoms of subthreshold severity; (b) brief limited intermittent psychotic symptoms (BLIPS), i.e., typical psychotic symptoms of short duration that remit spontaneously; or (b) genetic high risk with functional deterioration (GRD). Another conceptualization of risk focuses on basic symptoms; these are subtle subjective changes in perception, cognition and language that have been suggested to reflect cognitive disturbances present in the very early stages of the psychosis prodrome [4]. In the present paper, we use the term clinical high-risk (CHR) to refer to all of the above patients.

In relative terms, the risk of developing a full psychotic episode (‘transition to psychosis’) in CHR patients is increased by a factor of more than 400 compared to the general population [5] and reaches its peak within the first 2–3 years from diagnosis. In absolute terms, however, the majority of these patients will not experience a psychotic episode: Transition rates were initially calculated at about 36% at 3 years [6] and have been corrected downwards (20%) in newer meta-analyses [7]. To identify patients that are most likely to benefit from early intervention, a large body of research has been devoted to identifying characteristics that distinguish CHR patients who will later experience a psychotic transition from those who will not. In the era of precision medicine, knowledge of such characteristics is necessary to help guide intervention strategies based on individual patient prognosis and needs.

Given the above, we aimed to provide a consolidated evidence base to help identify promising foci for further research, to inform the development of prognostic models for individualized outcome prediction, and to guide the search for modifiable factors as treatment targets. Considering the fast-expanding field of psychosis prediction in CHR and the availability of several systematic reviews and meta-analyses, we deemed the format of an umbrella review most appropriate for our purpose. Thus, the aim of the present manuscript was to summarize evidence from systematic reviews and meta-analyses that investigated predictors of transition to psychosis in CHR patients, among characteristics and biomarkers assessed at baseline, i.e., at first diagnosis of CHR status. To the best of our knowledge, this is the first umbrella review with an exclusive focus on this topic.

## Methods

We conducted the review according to the Preferred Reporting Items for Systematic Reviews and Meta-Analysis (PRISMA) guidelines [8]. We submitted the protocol in the International Prospective Register of Systematic Reviews (PROSPERO) database on May 8, 2022 (registration number CRD42022331183). For a PRISMA Checklist, please refer to the Supplement (Table S1).

### Search

The search strategy was defined before data selection and extraction. For the literature search, we used Pubmed, Scopus, PsychInfo and Cochrane databases (last access date: 19.10.2022). The final search key (which was updated during the manuscript revision process) was [(‘clinical high risk’ OR ‘at-risk mental state’ OR ‘high risk’) AND (‘psychosis’ OR ‘psychotic disorder’ OR ‘schizophrenia’) AND (‘prediction’ OR ‘biomarker’ OR ‘associat*’)]; additional search terms or filters (depending on the database) were used to filter papers (a) published from March 1, 2012 to March 1, 2022; (b) written in English; and (c) registered as reviews, systematic reviews or meta-analyses. The exact search strings for each database are provided in the Supplement.

Additionally, the reference lists of included paper were scanned for further relevant reviews and meta-analyses.

### Eligibility criteria

Papers were included in the review if they met all of the following criteria:

1. publication in a peer-reviewed journal;
2. systematic review or meta-analysis (*study design*);
3. inclusion of studies that included patients with clinical high risk (3a. *Participants*) and investigated transition to psychosis (3b. *Outcome*) in relation to specific baseline predictors or biomarkers (3c. *Exposure*) using either cross-sectional comparisons between CHR with and without later transition, or a longitudinal design (3d. *Comparison*);
4. full-text available through university or public repositories, or by contacting the authors.

Gray literature, conference abstracts and narrative reviews were excluded. We also excluded papers investigating effects of interventions or longitudinal changes in predictors over time on transition.

### Study selection

**Fig. 1:**
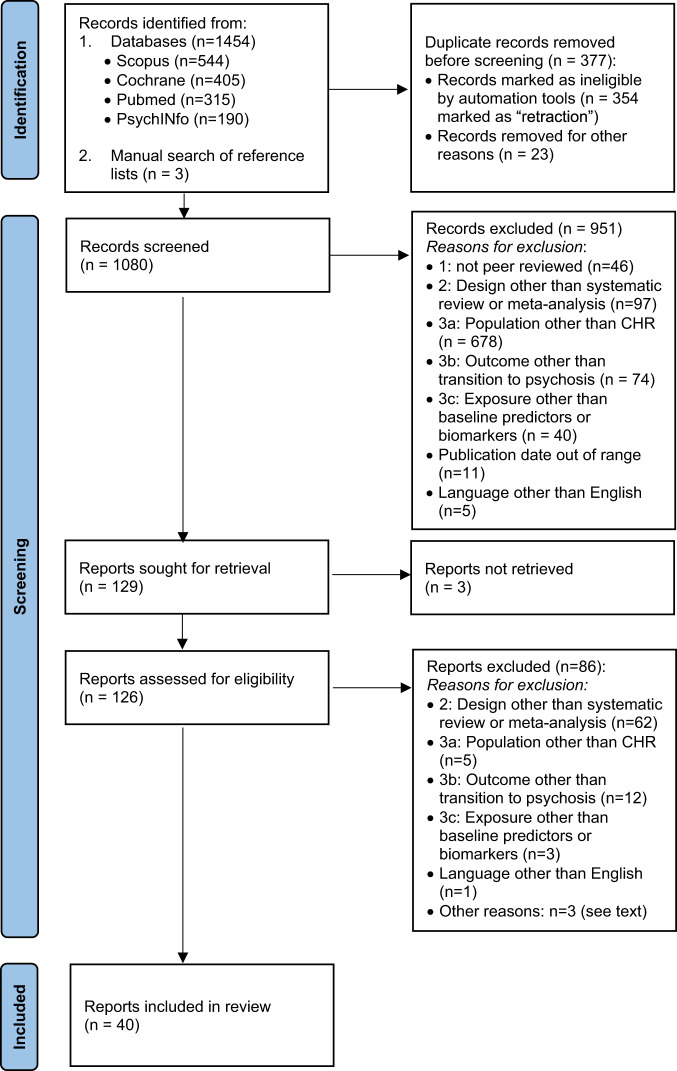
PRISMA data selection flow diagram. The number of studies identified/excluded at each step is indicated in brackets.

After articles were identified through the above outlined search strategy and records of duplicates were removed, titles and abstracts were screened by two independent authors (SE and MH). Records were excluded according to the above inclusion and exclusion criteria. All excluded and included articles were reviewed and potential discrepancies resolved by the first author. Of all included abstracts, full-text articles were assessed for eligibility by the first author; reasons for exclusion were reviewed by one of the two senior authors (see Fig. 1 for an overview of reasons for exclusion).

### Data extraction

The following variables were extracted by the first author for each paper if available: (1) name of the study; authors and publication year;(2) review design; (3) reviewed predictors or biomarkers; (4) number and citations of studies included in the review of each predictor/biomarker domain; (5) findings.

### Quality assessment

To assess the quality of included reviews and meta-analyses, we used the Checklist for Assessing the Methodological Quality of Systematic Reviews (AMSTAR), an 11-item tool which has been validated for systematic reviews of randomized controlled trials [9], but has been widely used for systematic reviews of other types of studies [10]. Each included paper was assessed by one of two authors (SE or MH), and a third author (CA) confirmed ratings and ensured that there were no systematic differences between the two raters.

### Data synthesis

We considered only data available from included systematic reviews and meta-analyses. We provide a narrative synthesis of results from all included reviews and meta-analyses. Given the large variability in topics and predictor domains, we chose to present and compare all reported findings without particular prioritization according to review recency or quality. To assist interpretation of findings, we indicate below the number of studies (*k*) included in each review (where available) as well as any significant overlaps in studies between reviews. We also highlight reviews of high quality.

## Results

### Search results

After removal of search duplicates, a total of 126 articles were screened for potential inclusion (see data selection flow diagram in Fig. 1). 40 articles qualified for inclusion (see Flow Diagram, Fig. 1); of these, 21 were systematic reviews and 19 were meta-analyses. An umbrella review by Fusar-Poli et al. [7] and a meta-analysis by Hager and Keshavan [11] were excluded because they assessed systematic reviews and meta-analyses rather than single studies; moreover, a meta-analysis by Sanfelici et al. [12] was excluded because it focused on performance of predictive models rather than single predictors of outcome. A list of included studies is provided in Table 1.

**Table 1:** List of included papers.

Authors	Year	Type of review	Investigated predictors (*k* = number of included studies)	AMSTAR score
Addington J, Case N, Saleem MM, Auther AM, Cornblatt BA, Cadenhead KS.	2014 [32]	Systematic review	Substance use (*k* = 10)	33%
Bodatsch M, Klosterkotter J, Muller R, Ruhrmann S.	2013 [46]	Systematic Review	fMRI (*k* = 5); electroencephalography (*k* = 10)	11%
Boldrini T, Tanzilli A, Pontillo M, Chirumbolo A, Vicari S, Lingiardi V.	2019 [63]	Systematic review	Clinical: personality disorders (*k* = 7)	100%
Bora E, Lin A, Wood SJ, Yung AR, McGorry PD, Pantelis C.	2014 [43]	Meta-analysis	Neurocognition (*k* = 11)	64%
Brew B, Doris M, Shannon C, Mulholland C.	2018 [17]	Systematic review	Environmental: trauma (*k* = 2)	67%
Catalan A, Salazar De Pablo G, Aymerich C, Damiani S, Sordi V, Radua J et al.	2021 [40]	Meta-analysis	Neurocognition (*k* = 22)	100%
Catalan A, Salazar de Pablo G, Serrano SV, Mosillo P, Baldwin H, Fernandez-Rivas A, et al.	2021 [26]	Meta-analysis	Symptoms (*k* = 1), neurocognition (*k* = 2) in children and adolescents	91%
Chaumette B, Kebir O, Mam-Lam-Fook C, et al.	2016 [60]	Meta-analysis	Inflammation: cortisol (*k* = 4)	64%
De Herdt A, Wampers M, Vancampfort D, De Hert M, Vanhees L, Demunter H et al.	2013 [39]	Meta-analysis	Neurocognitive (*k* = 10)	82%
Erickson M, Ruffle A, Gold J	2016 [53]	Meta-analysis	Electroencephalography (*k* = 5)	91%
Farris MS, Shakeel MK, Addington J.	2020 [35]	Meta-analysis	Cannabis use (*k* = 8)	73%
Fortea A, Batalla A, Radua J, van Eijndhoven P, Baeza I, Albajes-Eizagirre A et al.	2021 [49]	Meta-analysis	sMRI (*k* = 8)	91%
Fusar-Poli P, Deste G, Smieskova R, Barlati S, Yung AR, Howes O et al.	2012 [42]	Meta-analysis	Neurocognition (*k* = 7)	100%
Fusar-Poli P, Rocchetti M, Sardella A, Avila A, Brandizzi M, Caverzasi E, et al.	2015 [31]	Meta-analysis	Functioning (*k* = 10)	91%
Gogos A, Skokou M, Ferentinou E, Gourzis P.	2019 [37]	Systematic review	Nicotine use (*k* = 2)	67%
Hinney B, Walter A, Aghlmandi S, Andreou C, Borgwardt S.	2020 [48]	Meta-analysis	sMRI (*k* = 5)	91%
Izon E, Berry K, Law H, French P.	2018 [24]	Systematic review	Environmental: expressed emotion in the family (*k* = 1)	100%
Karanikas, E., Garyfallos, G., 2015.	2015 [59]	Systematic review	Inflammation: cortisol (*k* = 5)	33%
Khoury R, Nasrallah HA.	2018 [54]	Systematic review	Inflammation (*k* = 14)	56%
Kraan T, Velthorst E, Koenders L, Zwaart K, Ising HK, Van Den Berg D, et al.	2016 [34]	Meta-analysis	Cannabis use (*k* = 7)	91%
Malda A, Boonstra N, Barf H, de Jong S, Aleman A, Addington J et al.	2019 [15]	Meta-analysis	Sociodemographic, symptoms, functioning (*k* = 15)	100%
Misiak B, Bartoli F, Carrà G, Sta_czykiewicz B, G_adka A, Frydecka D et al.	2021 [56]	Meta-analysis	Inflammation (*k* = 4)	100%
Montemagni C, Bellino S, Bracale N, Bozzatello P, Rocca P.	2020 [19]	Systematic review	In multivariable models: symptoms (*k* = 14), functioning (*k* = 7), substane use (*k* = 2), sMRI (*k* = 3), EEG (*k* = 3), neurocognition (*k* = 2), sociodemographic (*k* = 5), inflammation (*k* = 1)	67%
Moore D, Castagnini E, Mifsud N, Geros H, Sizer H, Addington J et al.	2021 [16]	Systematic review	Sociodemographic: ethnicity (*k* = 4), migrant status (*k* = 3)	100%
O’Donoghue B, Roche E, Lane A.	2016 [20]	Systematic review	Environmental: social deprivation (*k* = 1)	78%
Oliver D, Reilly TJ, Baccaredda Boy O, Petros N, Davies C, Borgwardt S et al.	2020 [14]	Systematic review	Functioning, symptoms, sociodemographic, environmental, substance use (total *k* = 129)	100%
Park S, Miller BJ.	2020 [57]	Meta-analysis	Inflammation (*k* = 4)	64%
Peh OH, Rapisarda A, Lee J.	2019 [18]	Meta-analysis	Environmental: trauma (*k* = 5), bullying (*k* = 1)	100%
Perrottelli A, Giordano GM, Brando F, Giuliani L, Mucci A.	2021 [52]	Systematic review	Electroencephalography (*k* = 18)	67%
Pieters LE, Nadesalingam N, Walther S, van Harten PN.	2022 [65]	Systematic review	Motor signs (*k* = 7)	100%
Raballo A, Poletti M, Preti A.	2020 [67]	Meta-analysis	Other: antipsychotics (*k* = 16)	91%
Riecher-Rössler A, Studerus E	2017 [13]	Systematic review	Symptoms, functioning, substance use, neuroimaging, DUP, sociodemographic, genetic, environmental, neurocognition, hormones, inflammation, multivariable models (total *k* = 48)	44%
Romeo B, Petillion A, Martelli C, Benyamina A.	2020 [50]	Meta-analysis	MR spectroscopy (*k* = 6)	64%
Rosen M, Betz LT, Schultze-Lutter F, Chisholm K, Haidl TK, Kambeitz-Ilankovic L, et al.	2021 [21]	Systematic review	In Multivariable models: sociodemographic (*k* = 4), symptoms (*k* = 22), substance use (*k* = 2), functioning (*k* = 9), neurocognition (*k* = 5)	89%
Schiavone S, Trabace L.	2017 [55]	Systematic review	Inflammation (*k* = 2)	33%
Seabury RD, Cannon TD.	2020 [41]	Systematic review	fMRI (*k* = 1), neurocognition (*k* = 8), electroencephalography (*k* = 5)	56%
Tor J, Dolz M, Sintes A, Muñoz D, Pardo M, de la Serna E et al.	2018 [27]	Systematic review	Symptoms, neurocognition (*k* = 4)	78%
Treen D, Batlle S, Mollà L, Forcadell E, Chamorro J, Bulbena A et al.	2016 [47]	Systematic review	MR spectroscopy (*k* = 3)	44%
van der Steur SJ, Batalla A, Bossong MG.	2020 [33]	Systematic review	Cannabis use (*k* = 4)	67%
van Donkersgoed RJ, Wunderink L, Nieboer R, et al.	2015 [45]	Meta-analysis	Social cognition (*k* = 8)	82%

### Data synthesis

The 40 systematic reviews and meta-analyses we assessed reviewed a total of 272 original studies. Below, we present a synthesis of findings, separately for different types of predictors. A summary of main results can be found in Table 2. Reviews or meta-analyses with a high AMSTAR quality rating (>80%) are indicated by underscore in the text. Detailed quality ratings are provided in the Supplement (Table S2).

**Table 2:** Summary of evidence.

Predictor	N reviews	Evidence	Comments
Sociodemographic			
Age	4		Not significant on its own, but included in multivariable models
Sex	2		Included in multivariable models
Ethnicity	2		
Environmental			
Trauma	3		
Education	3		Not significant on its own, but included in multivariable models
Baseline living status	1		
Employment	2		
Parental socioeconomic status	1		
Perinatal complications	1		
Clinical			
Attenuated Psychotic Symptoms	4		Included in multivariable models
Negative symptoms	1		Included in multivariable models
Disorganized symptoms	2		Included in multivariable models
General symptoms	1		Included in multivariable models
Total symptoms	1		
Functioning	5		
Cannabis use	5		Cannabis use not predictive of transition, but severity of use potentially relevant
Other substance use	3		
Biomarkers			
Neurocognition	9		Unclear which domains are most predictive; included in multivariable models
Stress/Inflammation	4		Not significant on their own, but included in one multivariable model
Structural MRI	2		Significant effect for temporal and paracingulate cortical gray matter
MMN	4		Included in multivariable models
Other			
Antipsychotic exposure	1		
Handedness	1		

The number of squares refers to the number of total studies reviewed (1: 5–15 studies; 2: 16–30 studies; 3: >30 studies). Color code: green—evidence of a significant association; red—no significant or not conclusive evidence; orange—see comments. Effect sizes are reported in “Results”. Only domains with a total of ≥5 reviewed studies are included on the table.

#### Sociodemographic predictors

With respect to age at baseline, discrepant findings have been reported [13]; a recent meta-analysis concluded that the overall evidence for age was not significant (Oliver et al. [14], *k* = 61 studies). Still, age is often reported as a significant predictor in multivariable models [13], and was a significant predictor in a multivariable model derived from meta-analysis individual patient data from 15 studies, with higher age at baseline increasing transition risk (Malda et al. [15]).

Regarding sex, Oliver et al. [14] reported a weak association, with increased transition probability in males but small effect size (*k* = 66); similarly, female sex was included as a predictor reducing overall risk in the multivariable model developed in the above-mentioned individual patient data meta-analysis [15].

Ethnicity was investigated in one systematic review (Moore et al. [16], *k* = 4) and one meta-analysis (Oliver et al., *k* = 19 [14]; total *k* = 21). The effect of non-white ethnicity as a predictor of transition was non-significant in the meta-analysis by Oliver et al., while Moore et al. report divergent results regarding the comparison of different ethnic groups in terms of CHR transition rates.

#### Environmental predictors

Evidence for an effect of trauma on transition rates was significant but weak in a meta-analysis (Oliver et al. [14], 2021, *k* = 11, small effect size and moderate heterogeneity; see also Brew et al. [17], *k* = 2, and Peh et al. [18], *k* = 5, all overlapping with Oliver et al.). Similarly, there was weak evidence that baseline living status (*k* = 10) [14] and employment (Oliver et al. [14], *k* = 7; *k* = 1 also cited in Montemagni et al. [19]) may predict transition with a small-to-medium effect size. There are contradictory results regarding marital status (*k* = 2) [13] and no evidence of association for level of education (Riecher and Studerus [13], *k* = 8; Oliver et al. [14], *k* = 25; total *k* = 27), parental socioeconomic status (*k* = 14) [14], neighborhood-level social deprivation (O’Donoghue et al., *k* = 1) [20], urbanicity (*k* = 4) [14], migrant status (Oliver et al. [14], *k* = 2; Moore et al. [16], *k* = 3; total *k* = 4), brain injury (*k* = 2) [14] and perinatal complications (*k* = 6) [14], even though isolated studies have included education (*k* = 4) and urbanicity (*k* = 1) as significant predictors in multivariable models [19, 21].

Stress associated with perceived stigma was reported to be a significant predictor in two reviews (Montemagni et al. [19] and Riecher and Studerus [13]), although this result was based on a single study [22]. In contrast, overall evidence for stigma as a predictor of transition was found to be non-significant in the meta-analysis by Oliver et al. [14]; however, this conclusion was based only on two studies [22, 23], one of which was the above-mentioned study by Rüsch et al. [22].

Finally, a systematic review by Izon et al. [24] identified only one study that investigated the association of expressed emotion in the family with transition in CHR. The study in question [25] reported a significant association between transition and perceived irritability of the most important person in the social environment, but not other aspects of expressed emotion such as criticism and emotional overinvolvement.

#### Clinical predictors

##### Symptoms

Severity of attenuated psychotic symptoms at baseline is reported as a significant predictor of predictors in a meta-analysis that assessed findings in children and adolescents (Catalan et al. [26], *k* = 1), and was one of only two predictors that achieved a highly suggestive evidence level in a meta-analysis by Oliver et al. [14] (*k* = 49), albeit with a small effect size. This conclusion is consistent with earlier reviews [13, 27]. Basic symptoms, on the other hand, were reported to be a significant predictor of transition by Tor et al. [27] based on one single study [28], but a more recent meta-analysis by Oliver et al. [14] found no association based on two further studies [29, 30]; the scarcity of evidence probably contributes to this discrepancy.

Oliver et al. [14] also reported suggestive evidence for negative symptoms (*k* = 49), but only weak evidence for disorganized/cognitive symptoms (*k* = 18; see also Tor et al. [27], *k* = 2), general symptoms (*k* = 21) and total symptoms (*k* = 21); in all cases, the effect sizes reported were small. Symptoms belong to the predictors more frequently assessed in multivariable models [19, 21]; the majority of these models include (attenuated) positive psychotic symptoms such as auditory hallucinations or delusions (*k* = 14) [19, 21], but some studies have also included negative (*k* = 5) [21], disorganized (*k* = 10) [19, 21] or depressive symptoms (*k* = 2) [19], or sleep disturbances (*k* = 2) [19, 21]. Language abnormalities such as illogical thinking, poverty of content and reduced referential cohesion might also be relevant for prediction of transition according to Tor et al. [27], although this conclusion is based only on one study.

Apart from the type of symptoms as described above, duration of symptoms at baseline was included as a predictor of transition in three multivariable models reviewed by Montemagni et al. [19] and Rosen et al. [21].

##### Functioning

All five papers assessing this domain (two meta-analyses [14, 31] and three systematic reviews [13, 19, 21]) report low levels of global functioning at baseline (assessed with the GAF or SOFAS) as a predictor of transition. Fusar-Poli et al. [31] reported a small-to-medium magnitude for this effect and moderate heterogeneity of results in the 10 studies included in their meta-analysis; a more recent meta-analysis by Oliver et al. [14] judged the level of evidence for functioning as a predictor of transition as highly suggestive and a small effect size, based on 49 studies.

#### Substance use

Two systematic reviews and three meta-analyses (Addington et al. [32], *k* = 10; van der Steur et al. [33], *k* = 4; Kraan et al. [34], *k* = 7; Farris et al. [35], *k* = 8; Oliver et al. [14], *k* = 23; total k = 31) found no significant association of cannabis use with transition. However, one study (Valmaggia et al., cited in van der Steur et al. [33], Kraan et al. [34], Oliver et al. [14], and Farris et al. [35]) reported higher transition rates in frequent compared to non-frequent users. Another study identified severity of use as a potentially relevant factor (McHugh et al. [36], cited in van der Steur et al. [33] and Riecher and Studerus [13]); this variable considers substance abuse characteristics beyond frequency of use, specifically the subjective need for the substance, impaired capacity to control use, impaired capacity to stop use, social problems and risk-taking behavior associated with use [36]. In line with this finding, the presence of cannabis *dependence* was a significant predictor of transition in one meta-analysis (*k* = 5) [34], an effect of moderate magnitude. Although younger age of onset of cannabis use was found to result in younger age of psychosis symptom onset (*k* = 1) [32], findings regarding the relevance of age at onset of use for transition probability have been discrepant (*k* = 2) [33]. Moreover, Farris et al. reported that age, male gender and continent were not statistically significant factors contributing to heterogeneity between studies.

Use of other substances including tobacco (Oliver et al. [14], *k* = 10; Gogos et al. [37], *k* = 2; total *k* = 10) and alcohol (*k* = 10) [14] has not been found to predict transition, even though substance use has been included in one influential predictive model of transition (Cannon et al. [38] cited in Montemagni et al. [19]). One study even reported reduced alcohol use in patients who later transitioned into psychosis, but its authors interpreted this effect as a reflection of social withdrawal (Buchy et al., cited in Montemagni et al. [19]).

#### Neurocognition

Some multivariable predictive models of transition have included neurocognitive performance as significant predictors (*k* = 2) [19], although the type of domains/tests included varies. Three meta-analyses [26, 39, 40] and four systematic reviews [13, 19, 21, 27, 41] have investigated neurocognitive performance at baseline as a predictor of transition. Three early meta-analyses (de Herdt et al. [39], *k* = 10; Fusar-Poli et al. [42], *k* = 7; Bora et al. [43], *k* = 11; total *k* = 17) yielded divergent findings: De Herdt et al. reported significant effects only for working memory and visual learning, with a small effect size; Fusar-Poli et al. [42] additionally reported a significant association of verbal fluency and verbal memory, as well as IQ with later transition; while Bora et al. [43] reported that patients with later transition showed lower IQ and impairment in all cognitive domains except sustained attention and with small-to-medium effect sizes, although individual tasks (TMT-A and letter-number sequencing) were not associated with transition. A more recent meta-analysis (Catalan et al. [40], *k* = 22; overlap of 4 studies with the above three meta-analyses) found that deficits in verbal memory (also reported as a significant predictor by Seabury and Cannon [41], with an effect size of 0.39, *k* = 8), visual memory, executive function, processing speed, attention/vigilance and IQ were associated with later transition with moderate heterogeneity and a small-to medium effect size, although the magnitude of effect was dependent on the exact paradigm used to assess each cognitive domain [40]. The same meta-analysis found no significant effect for verbal fluency (*k* = 4) and working memory (*k* = 5) [40]. The latter finding is in contrast to previous systematic reviews (Seabury and Cannon [41], *k* = 4; Riecher and Studerus [13], *k* = 2) that reported working memory as a predictor of transition, and to its inclusion as a significant predictor in multivariable models (*k* = 3) [21]. The discrepancy in findings might be explained by the rather small magnitude of the effect of working memory, by differences in the paradigms used and in the statistical methods applied, or by differences in sampling [41]: For example, working memory lost its significance as a predictor of transition after controlling for demographic characteristics in a study by Carrion et al. [44] (cited in Seabury and Cannon [41]).

Social cognition has also been investigated as a predictor of transition. Findings in this field have been summarized by van Donkersgoed et al. [45] in a systematic review: The evidence they present does not support an association of theory of mind (*k* = 4), prosodic affect recognition (*k* = 2), social perception (*k* = 1) and attributional style (*k* = 1) with later transition. Results regarding facial affect recognition were mixed, with two negative studies and two further studies reporting abnormal performance in patients with later transition, but opposite patterns of emotion mislabeling [45].

#### Structural and functional MRI

Neuroimaging represents a very active research area in the search of biomarkers for transition. Our search identified five systematic reviews [13, 19, 41, 46, 47] and three meta-analyses [48–50]; however, all were devoted to specific, circumscribed questions. For more comprehensive reviews of the literature in this field, we refer to reader to a recent narrative review by our group [51] and to a comprehensive umbrella review by Hager and Keshavan [11].

Bodatsch et al. [46] (*k* = 5) and Seabury and Cannon [41] (*k* = 1) reviewed fMRI studies of cognitive processing. They both report increased activation in task-related and subcortical areas in patients with later transition, such as increased activity in temporal, language, precentral, caudate and striate regions during language processing [46]; increased activity in frontal regions, the brainstem and the hippocampus (as well as increased midbrain-prefrontal connectivity) during verbal fluency tasks [46]; and increased activity in left frontal, left inferior parietal and right temporal areas during verbal memory retrieval [41]. Altogether, these findings appear relatively unspecific in terms of functional neuroanatomy and are based on few studies, often with overlapping patient populations. Regarding resting-state fMRI, Riecher and Studerus [13] reported reduced connectivity in the salience network and aberrant thalamocortical connectivity, each investigated in one study.

Three papers addressed structural MRI findings. Of these, Montemagni et al. [19] reported that volumetric changes in prefrontal perisylvian and subcortical structures were included in two predictive models of transition, and that a further study used multivariate pattern classification based on structural MRI data. In contrast, a single-voxel meta-analysis of cortical gray matter by Fortea et al. [49] (*k* = 8) reported reductions in the right temporal lobe and superior and middle temporal gyrus, as well as right ACC and paracingulate gyrus in CHR patients with later transition compared to those without, with a small-to-medium effect size. A meta-analysis by Hinney et al. [48], on the other hand, focused on hippocampus size as a potential predictor of transition (*k* = 5); the authors found small, non-significant differences between patients with and without later transition in the left and right hippocampus, and high heterogeneity in findings regarding the left hippocampus.

Finally, magnetic resonance spectroscopy findings were addressed by a meta-analysis (Romeo et al. [50], *k* = 6) and a systematic review (Treen et al. [47], *k* = 1, included in Romeo et al). Given the considerable variation in investigated metabolites and brain regions, many reported findings were based on single studies. The most consistent findings concerned N-acetylaspartate and choline in the medial temporal lobe (*k* = 3) and the dorsolateral prefrontal cortex (*k* = 2), and Glx (i.e., the combined signal of glutamate and glutamine) in the medial temporal lobe (*k* = 3), for which no association with transition was observed.

#### Electrophysiology

Several electroencephalography (EEG)-based markers have been investigated as predictors of transition in clinical high-risk patients. The most consistent finding is mismatch negativity (MMN), an event-related potential (ERP) elicited by infrequent deviant tones in a sequence of standard tones. In all included reviews, the majority of reviewed studies report reduced MMN amplitude in patients with compared to those without later transition (Bodatsch et al. [46], *k* = 4; Perrottelli et al. [52], *k* = 6; Seabury and Cannon [41], *k* = 5; total *k* = 6). This association was confirmed in a recent meta-analysis by Ericson et al. [53] (*k* = 5) and is more prominent for MMN in response to duration (dMMN, *k* = 6) than to pitch (pMMN, k = 2) deviants [52].

Another frequent research subject is the P3 component, elicited in response of low probability target stimuli in oddball paradigms. Evidence regarding the P3 with respect to transition is rather inconsistent (*k* = 2) [46], which may be due to different paradigms used: The majority of studies investigating the subcomponent P3b (elicited by target stimuli as described above) found it to be reduced in amplitude in patients with later transition (*k* = 5) [52], whereas results regarding the P3a (elicited by infrequent distractor stimuli that require no response [52]) are mixed, with most studies finding no association with later transition (*k* = 3).

Results regarding other ERP markers such as prepulse inhibition (i.e., a weak pre-stimulus inhibiting an ensuing reflex-like response; *k* = 1) [46] and sensory gating (i.e., the relative suppression in a quick succession of sensory events; *k* = 3) [46] are mixed. Other resting-state EEG markers such as scalp or source power, or synchronicity, in specific frequency bands, have also been investigated in a few studies but yielded inconsistent results (Perrottelli et al. [52], *k* = 4; Riecher and Studerus [13], *k* = 3).

#### Stress hormones and inflammatory biomarkers

Evidence regarding inflammatory biomarkers was reviewed in two systematic reviews (Khoury and Nasrallah [54], *k* = 14; Schiavone and Trabace [55], *k* = 2; total *k* = 14) and two meta-analyses (Misiak et al. [56], *k* = 4; Park and Miller [57], *k* = 4; total *k* = 4, all included in the review by Khoury and Nasrallah [54]). Overall, there is no significant evidence linking inflammatory, pro-inflammatory or anti-inflammatory cytokines to later transition [54, 56, 57]; the same holds for acute-phase proteins (CRP and fibrinogen), although one small study reported reduced albumin plasma levels in patients with later transition [58] (cited in Khoury and Nasrallah [54]). Similarly, most studies investigating cortisol levels in plasma or saliva have not found differences with respect to later transition (Khoury and Nasrallah [54], *k* = 5; Karanikas and Garyfallos [59], *k* = 4; total *k* = 7), and the effect of cortisol as a predictor was found to be non-significant in a recent meta-analysis (Chaumette et al. [60], *k* = 5). Evidence regarding other aspects of cortisol regulation is limited, with one small study observing lower cortisol peak levels after dexamethasone suppression [61] (cited in Karanikas and Garyfallos [59] and Khoury and Nasrallah [54]), while two further studies reported opposite patterns (increase vs. decrease) of cortisol awakening response in patients with later transition [54]. However, a classifier that was developed within the NAPLS study using greedy algorithm analysis of 117 potential biomarkers included four cytokines (IL-1β, IL-7, IL-8 and chemokine-ligand 8), as well as cortisol and matrix metallo-proteinase(MMP)-7 as predictors of transition among a final set of 15 selected biomarkers. [19, 54]

Finally, one study [13] (reviewed by Riecher and Studerus [13]) reported increased prolactin levels in patients with later transition, although it is unclear whether these represent a stress-related epiphenomenon, or contribute to the emergence of psychosis via pro-inflammatory or cognitive-mediated pathways [62].

#### Other predictors

Boldrini et al. [63] systematically reviewed evidence on the link of personality characteristics to later psychotic transition. The authors reported mixed results regarding schizotypal personality disorder (*k* = 3) and note that studies that found an association investigated samples with a greater mean age or implemented a longer follow-up period. Their interpretation was that schizotypal personality disorder represents a ‘distal’ trait factor, i.e., temporally more remote from the development of psychosis, and thus more important in the long term. One study [64] (cited in Boldrini et al. [63]) observed a link between schizoid features at baseline and later transition, although this effect was weak. Finally, borderline personality characteristics did not predict transition in the few studies (*k* = 2) that investigated this predictor, although no definite conclusions can be made because of the small sample sizes and/or the use of self-rating scales rather than diagnostic interviews [63].

Pieters et al. [65] summarized evidence on motor abnormalities as predictors of transition in a systematic review. They report mixed results with respect to neurological soft signs, but increased baseline dyskinesia in patients with later transition (*k* = 3); moreover, one study found that motor dysfunction rated in the scale of prodromal symptoms was associated with later transition [66] (cited in Pieters et al. [65]).

Exposure to antipsychotic at the timepoint of CHR diagnosis was investigated in a meta-analysis by Raballo et al. [67] (*k* = 16). The authors reported that CHR under treatment with antipsychotics at baseline had a higher risk of transition, but the magnitude of effect was small, there was high heterogeneity between studies and the association was not significant in the random-effects model.

Finally, Oliver et al. [14] report weak evidence for an association of transition with right-handedness (*k* = 16) with a small effect size, and no significant evidence linking height (*k* = 5) or BMI (*k* = 3) to later transition.

#### Multivariable models with combined predictors

In recent years, a growing number of studies have developed predictive models of transition based on a combination of different predictors. Most of these combine symptom patterns with further predictors such as sociodemographic/environmental predictors, neurocognition, electrophysiological or serum biomarkers, in the hope of improving predictive performance compared to symptoms alone. [13, 19, 21] Although many of these models achieve good discrimination performance, they differ widely in their selection of included variables and most of them have not been independently validated, making their generalizability difficult to assess. In an innovative approach, Rosen et al. [21] identified 22 prediction models of transition by systematic review and evaluated their performance in an independent dataset of 173 CHR patients. Discrimination performance varied widely across models, with only 13 models performing above chance and only two models achieving acceptable discrimination (defined by the statistical criterion of area under the curve (AUC), with AUC ≥ 0.70, indicating a model that identifies 70% οf transitions correctly) [21].

In an alternative approach, Malda et al. [15] used data from a total of 1676 individual patients from 15 studies to create an individualized prognostic model of transition in CHR based on simple variables assessed at baseline. Significant predictors in the model were sex, age, the type of risk (genetic risk with functional deterioration, attenuated psychotic symptoms, or brief limited intermittent psychotic symptoms), functioning, and negative and positive psychotic symptom severity. The model was evaluated using internal-external cross-validation (i.e., ‘leave-one-study-out’), which resulted in performance that was higher than chance, but moderate.

## Discussion

The present umbrella review aimed to provide an overview of evidence regarding predictors of transition among characteristics present at baseline in CHR patients. Predictors most consistently associated with later transition were attenuated psychotic symptoms and functioning. There is also adequate support for negative symptoms and neuropsychological deficits, especially verbal memory, as predictors of transition. Further, there are indications for a potential role of some sociodemographic (sex, age) and environmental factors (living status, employment, perinatal complications), trauma, cannabis dependence, disorganized symptoms and the electrophysiological marker of the MMN. However, the evidence base for all of these latter factors is less extensive (i.e., based on single reviews or a smaller number of studies).

Our search identified 40 systematic reviews, which assessed a total of 272 studies, as well as 2 umbrella reviews and 62 further narrative reviews published in the past 10 years. More than a third of included reviews (*k* = 16) were published in the last 2 years, reflecting a rather active, though specialized, field of research. In light of this, the most notable finding of our review is the relative paucity of associations that are sufficiently reliable to influence clinical practice. Most predictors have been assessed in a few studies with small sample sizes. This is a general shortcoming of prediction research in CHR patients, given that risk is defined only in help-seeking populations (thus limiting recruitment possibilities), while heterogeneous populations and relatively infrequent transition events pose a challenge on generalizability. Such an example of generalizability issues can be found in an analysis of machine learning models for schizophrenia [68], which observed diminishing diagnostic accuracy with increasing sample size, presumably due to overfitting in models derived from small samples.

The diversity of methods and its effects on study comparability is another issue that limits interpretation of findings. With respect to variable selection, the results of this diversity are particularly evident in prognostic models investigating symptoms as predictors of transition, with some including broader categories such as positive symptoms and disorganized communication, while others include more fine-grained items such as unusual thought content and suspiciousness, or illogical thinking, which differ between studies [19, 21]. Similarly, coding of variables such as ethnicity varies between studies, with some assessing the effect of non-white ethnicity, while others take a more differentiated view of ethnic groups [14]; the impact of these differences on findings has not been systematically studied. Another factor that might affect findings are varying referral and/or recruitment practices and assessment standards across sites [51]. For example, Sanfelici et al. [12] note that American studies have used younger samples than European studies. Therefore, broad replication of samples is of utmost importance. This is nicely demonstrated by performance of a risk calculator by Cannon et al. [69] in different CHR populations: The risk calculator achieved adequate discrimination (71%) in the original North American sample [69], and comparable performance (79%) in an external validation sample from another project that recruited patients in the United States of America [70]. However, replication in a Chinese sample resulted in much lower discrimination performance (63%) [71]. A final factor that affects reported findings are analysis methods. For example, cross-sectional comparisons at baseline between patients with and without later transition have produced different results (e.g., regarding age) than multivariable models, which take into account correlations between predictors. On the other hand, performance and generalizability of multivariate models depend substantially on the statistical approach used [12].

Based on the above, a strategical issue emerges: How is the field to proceed in order to identify predictors and generate prognostic models that are sufficiently reliable to inform clinical practice? An obvious inference is that there is urgent need for coordinated action and harmonization of methods across different sites, in order to achieve adequate power and generalizability through large samples and robust validation procedures. A step in this direction was made recently in the form of HARMONY (National Institute of Mental Health’s Harmonization of At-Risk Multisite Observational Networks for Youth), a collaboration of four large-scale projects that address individualized prediction in psychotic disorders and CHR (NAPLS, PNC [72], PSYSCAN and PRONIA), which aims to enable joint analyses of their data and cross-validation of their results. Alternative strategies are also emerging: One such promising approach is transdiagnostic prediction of outcomes other than psychotic transition such as functioning, severity across different symptom dimensions, or treatment response. This approach has the advantage of addressing the multidimensional symptomatology of CHR patients, most of whom present with at least one other formal psychiatric diagnosis and have high psychiatric morbidity and functional impairment in the long term irrespective of transition. First studies have adopted such a perspective, focusing on (transdiagnostic) prediction of outcomes other than transition [73–75]. Another promising avenue is the application of new techniques to better understand performance of prediction models, such as explainable artificial intelligence, which analyses the contributions of specific variables at the level of the individual and is able to detect potential biases in models [76].

Some limitations of our review need to be acknowledged. First, we chose to represent all findings without prioritizing according to specific criteria to better reflect the heterogeneous field of CHR prediction research, as described above. We tried to minimize the risk of drawing inferences based on outdated or low-quality findings by providing details about the number of studies included and the methodological quality of each review or meta-analysis. Second, our review only included papers investigating baseline predictors of transition, as these are potentially relevant for stratification of patients to interventions of different intensity. However, dynamic changes in certain predictors such as functioning or neurocognitive deficits [77, 78] are also important for predicting an impending transition and should be considered in further reviews.

## Conclusions

After more than 30 years of clinical high-risk (CHR) research, few findings regarding prediction of transition in CHR patients are supported by a robust evidence base. The present umbrella review identified high-level evidence supporting clinical characteristics such as attenuated psychotic symptoms, negative symptoms and functioning, neurocognitive deficits (particularly verbal memory) and an electrophysiological marker (mismatch negativity) at baseline as the best-established predictors of later transition in CHR patients. Further predictors have shown promise but need to be investigated in future studies and refined prediction models. Large samples and harmonization of methods are necessary to overcome the limitations of a currently still very heterogeneous field of research.

### Supplementary information

Supplemental Material

Prisma Checklist

## References

[1] Hafner H, Maurer K, Loffler W, an der Heiden W, Munk-Jorgensen P, Hambrecht M (1998). The ABC Schizophrenia Study: a preliminary overview of the results. Soc Psychiatry Psychiatr Epidemiol.

[2] Oliver D, Davies C, Crossland G, Lim S, Gifford G, McGuire P (2018). Can we reduce the duration of untreated psychosis? A systematic review and meta-analysis of controlled interventional studies. Schizophr Bull.

[3] Fusar-Poli P, Cappucciati M, Rutigliano G, Lee TY, Beverly Q, Bonoldi I (2016). Towards a standard psychometric diagnostic interview for subjects at ultra high risk of psychosis: CAARMS versus SIPS. Psychiatry.

[4] Schultze-Lutter F, Michel C, Schmidt SJ, Schimmelmann BG, Maric NP, Salokangas RK (2015). EPA guidance on the early detection of clinical high risk states of psychoses. Eur Psychiatry.

[5] Fusar-Poli P, Tantardini M, De Simone S, Ramella-Cravaro V, Oliver D, Kingdon J (2017). Deconstructing vulnerability for psychosis: meta-analysis of environmental risk factors for psychosis in subjects at ultra high-risk. Eur Psychiatry.

[6] Fusar-Poli P, Bonoldi I, Yung AR, Borgwardt S, Kempton MJ, Valmaggia L (2012). Predicting psychosis: meta-analysis of transition outcomes in individuals at high clinical risk. Arch Gen Psychiatry.

[7] Fusar-Poli P, Salazar de Pablo G, Correll CU, Meyer-Lindenberg A, Millan MJ, Borgwardt S (2020). Prevention of psychosis: advances in detection, prognosis, and intervention. JAMA Psychiatry.

[8] Moher D, Liberati A, Tetzlaff J, Altman DG, Group P (2009). Preferred reporting items for systematic reviews and meta-analyses: the PRISMA statement. PLoS Med.

[9] Shea BJ, Hamel C, Wells GA, Bouter LM, Kristjansson E, Grimshaw J (2009). AMSTAR is a reliable and valid measurement tool to assess the methodological quality of systematic reviews. J Clin Epidemiol.

[10] Pieper D, Koensgen N, Breuing J, Ge L, Wegewitz U (2018). How is AMSTAR applied by authors - a call for better reporting. BMC Med Res Methodol.

[11] Hager BM, Keshavan MS (2015). Neuroimaging biomarkers for psychosis. Cur Behav Neurosci Rep.

[12] Sanfelici R, Dwyer DB, Antonucci LA, Koutsouleris N (2020). Individualized diagnostic and prognostic models for patients with psychosis risk syndromes: a meta-analytic view on the state of the art. Biol Psychiatry.

[13] Riecher-Rössler A, Studerus E (2017). Prediction of conversion to psychosis in individuals with an at-risk mental state: A brief update on recent developments. Curr Opin Psychiatry.

[14] Oliver D, Reilly TJ, Baccaredda Boy O, Petros N, Davies C, Borgwardt S (2020). What causes the onset of psychosis in individuals at clinical high risk? A meta-analysis of risk and protective factors. Schizophr Bull.

[15] Malda A, Boonstra N, Barf H, de Jong S, Aleman A, Addington J (2019). Individualized prediction of transition to psychosis in 1,676 individuals at clinical high risk: development and validation of a multivariable prediction model based on individual patient data meta-analysis. Front Psychiatry.

[16] Moore D, Castagnini E, Mifsud N, Geros H, Sizer H, Addington J (2021). The associations between migrant status and ethnicity and the identification of individuals at ultra-high risk for psychosis and transition to psychosis: a systematic review. Soc Psychiatry Psychiatr Epidemiol.

[17] Brew B, Doris M, Shannon C, Mulholland C (2018). What impact does trauma have on the at-risk mental state? A systematic literature review. Early Inter Psychiatry.

[18] Peh OH, Rapisarda A, Lee J (2019). Childhood adversities in people at ultra-high risk (UHR) for psychosis: a systematic review and meta-analysis. Psychol Med.

[19] Montemagni C, Bellino S, Bracale N, Bozzatello P, Rocca P (2020). Models predicting psychosis in patients with high clinical risk: a systematic review. Front Psychiatry.

[20] O’Donoghue B, Roche E, Lane A (2016). Neighbourhood level social deprivation and the risk of psychotic disorders: a systematic review. Soc Psychiatry Psychiatr Epidemiol.

[21] Rosen M, Betz LT, Schultze-Lutter F, Chisholm K, Haidl TK, Kambeitz-Ilankovic L (2021). Towards clinical application of prediction models for transition to psychosis: a systematic review and external validation study in the PRONIA sample. Neurosci Biobehav Rev.

[22] Rusch N, Heekeren K, Theodoridou A, Muller M, Corrigan PW, Mayer B (2015). Stigma as a stressor and transition to schizophrenia after one year among young people at risk of psychosis. Schizophr Res.

[23] Salokangas RK, Patterson P, Heinimaa M, Svirskis T, From T, Vaskelainen L (2012). Perceived negative attitude of others predicts transition to psychosis in patients at risk of psychosis. Eur Psychiatry.

[24] Izon E, Berry K, Law H, French P (2018). Expressed emotion (EE) in families of individuals at-risk of developing psychosis: a systematic review. Psychiatry Res.

[25] Haidl T, Rosen M, Schultze-Lutter F, Nieman D, Eggers S, Heinimaa M (2018). Expressed emotion as a predictor of the first psychotic episode - results of the European prediction of psychosis study. Schizophr Res.

[26] Catalan A, de Pablo GS, Serrano JV, Mosillo P, Baldwin H, Fernandez-Rivas A (2021). Annual research review: prevention of psychosis in adolescents-systematic review and meta-analysis of advances in detection, prognosis and intervention. J Child Psychol Psychiatry.

[27] Tor J, Dolz M, Sintes A, Muñoz D, Pardo M, de la Serna E (2018). Clinical high risk for psychosis in children and adolescents: a systematic review. Eur Child Adolesc Psychiatry.

[28] Ziermans T, de Wit S, Schothorst P, Sprong M, van Engeland H, Kahn R (2014). Neurocognitive and clinical predictors of long-term outcome in adolescents at ultra-high risk for psychosis: a 6-year follow-up. PLoS ONE.

[29] Bang M, Park JY, Kim KR, Lee SY, Song YY, Kang JI (2019). Psychotic conversion of individuals at ultra-high risk for psychosis: The potential roles of schizotypy and basic symptoms. Early Inter Psychiatry.

[30] Mechelli A, Lin A, Wood S, McGorry P, Amminger P, Tognin S (2017). Using clinical information to make individualized prognostic predictions in people at ultra high risk for psychosis. Schizophr Res.

[31] Fusar-Poli P, Rocchetti M, Sardella A, Avila A, Brandizzi M, Caverzasi E (2015). Disorder, not just state of risk: meta-analysis of functioning and quality of life in people at high risk of psychosis. Br J Psychiatry.

[32] Addington J, Case N, Saleem MM, Auther AM, Cornblatt BA, Cadenhead KS (2014). Substance use in clinical high risk for psychosis: a review of the literature. Early Interv Psychiatry.

[33] van der Steur SJ, Batalla A, Bossong MG (2020). Factors moderating the association between cannabis use and psychosis risk: a systematic review. Brain Sci.

[34] Kraan T, Velthorst E, Koenders L, Zwaart K, Ising HK, van den Berg D (2016). Cannabis use and transition to psychosis in individuals at ultra-high risk: review and meta-analysis. Psychol Med.

[35] Farris MS, Shakeel MK, Addington J (2020). Cannabis use in individuals at clinical high-risk for psychosis: a comprehensive review. Soc Psychiatry Psychiatr Epidemiol.

[36] McHugh MJ, McGorry PD, Yung AR, Lin A, Wood SJ, Hartmann JA (2017). Cannabis-induced attenuated psychotic symptoms: implications for prognosis in young people at ultra-high risk for psychosis. Psychol Med.

[37] Gogos A, Skokou M, Ferentinou E, Gourzis P (2019). Nicotine consumption during the prodromal phase of schizophrenia-a review of the literature. Neuropsychiatr Dis Treat.

[38] Cannon TD, Cadenhead K, Cornblatt B, Woods SW, Addington J, Walker E (2008). Prediction of psychosis in youth at high clinical risk: a multisite longitudinal study in North America. Arch Gen Psychiatry.

[39] De Herdt A, Wampers M, Vancampfort D, De Hert M, Vanhees L, Demunter H (2013). Neurocognition in clinical high risk young adults who did or did not convert to a first schizophrenic psychosis: a meta-analysis. Schizophr Res.

[40] Catalan A, Salazar De Pablo G, Aymerich C, Damiani S, Sordi V, Radua J (2021). Neurocognitive functioning in individuals at clinical high risk for psychosis: a systematic review and meta-analysis. JAMA Psychiatry.

[41] Seabury RD, Cannon TD (2020). Memory impairments and psychosis prediction: a scoping review and theoretical overview. Neuropsychol Rev.

[42] Fusar-Poli P, Deste G, Smieskova R, Barlati S, Yung AR, Howes O (2012). Cognitive functioning in prodromal psychosis: a meta-analysis. Arch Gen Psychiatry.

[43] Bora E, Lin A, Wood SJ, Yung AR, McGorry PD, Pantelis C (2014). Cognitive deficits in youth with familial and clinical high risk to psychosis: a systematic review and meta-analysis. Acta Psychiatr Scand.

[44] Carrion RE, Walder DJ, Auther AM, McLaughlin D, Zyla HO, Adelsheim S (2018). From the psychosis prodrome to the first-episode of psychosis: no evidence of a cognitive decline. J Psychiatr Res.

[45] van Donkersgoed RJ, Wunderink L, Nieboer R, Aleman A, Pijnenborg GH (2015). Social cognition in individuals at ultra-high risk for psychosis: a meta-analysis. PLoS ONE.

[46] Bodatsch M, Klosterkotter J, Muller R, Ruhrmann S (2013). Basic disturbances of information processing in psychosis prediction. Front Psychiatry.

[47] Treen D, Batlle S, Mollà L, Forcadell E, Chamorro J, Bulbena A (2016). Are there glutamate abnormalities in subjects at high risk mental state for psychosis? A review of the evidence. Schizophr Res.

[48] Hinney B, Walter A, Aghlmandi S, Andreou C, Borgwardt S (2020). Does hippocampal volume predict transition to psychosis in a high-risk group? A meta-analysis. Front Psychiatry.

[49] Fortea A, Batalla A, Radua J, van Eijndhoven P, Baeza I, Albajes-Eizagirre A (2021). Cortical gray matter reduction precedes transition to psychosis in individuals at clinical high-risk for psychosis: a voxel-based meta-analysis. Schizophr Res.

[50] Romeo B, Petillion A, Martelli C, Benyamina A (2020). Magnetic resonance spectroscopy studies in subjects with high risk for psychosis: a meta-analysis and review. J Psychiatr Res.

[51] Andreou C, Borgwardt S (2020). Structural and functional imaging markers for susceptibility to psychosis. Mol Psychiatry.

[52] Perrottelli A, Giordano GM, Brando F, Giuliani L, Mucci A (2021). EEG-based measures in at-risk mental state and early stages of schizophrenia: a systematic review. Front Psychiatry.

[53] Erickson MA, Ruffle A, Gold JM (2016). A meta-analysis of mismatch negativity in schizophrenia: from clinical risk to disease specificity and progression. Biol Psychiatry.

[54] Khoury R, Nasrallah HA (2018). Inflammatory biomarkers in individuals at clinical high risk for psychosis (CHR-P): State or trait?. Schizophr Res.

[55] Schiavone S, Trabace L (2017). Inflammation, stress response, and redox dysregulation biomarkers: Clinical outcomes and pharmacological implications for psychosis. Front Psychiatry.

[56] Misiak B, Bartoli F, Carrà G, Stańczykiewicz B, Gładka A, Frydecka D (2021). Immune-inflammatory markers and psychosis risk: A systematic review and meta-analysis. Psychoneuroendocrinology.

[57] Park S, Miller BJ (2020). Meta-analysis of cytokine and C-reactive protein levels in high-risk psychosis. Schizophr Res.

[58] Labad J, Stojanovic-Perez A, Montalvo I, Sole M, Cabezas A, Ortega L (2015). Stress biomarkers as predictors of transition to psychosis in at-risk mental states: roles for cortisol, prolactin and albumin. J Psychiatr Res.

[59] Karanikas E, Garyfallos G (2015). Role of cortisol in patients at risk for psychosis mental state and psychopathological correlates: a systematic review. Psychiatry Clin Neurosci.

[60] Chaumette B, Kebir O, Mam-Lam-Fook C, Morvan Y, Bourgin J, Godsil BP (2016). Salivary cortisol in early psychosis: New findings and meta-analysis. Psychoneuroendocrinology.

[61] Thompson KN, Berger G, Phillips LJ, Komesaroff P, Purcell R, McGorry PD (2007). HPA axis functioning associated with transition to psychosis: combined DEX/CRH test. J Psychiatr Res.

[62] Labad J (2019). The role of cortisol and prolactin in the pathogenesis and clinical expression of psychotic disorders. Psychoneuroendocrinology.

[63] Boldrini T, Tanzilli A, Pontillo M, Chirumbolo A, Vicari S, Lingiardi V (2019). Comorbid personality disorders in individuals with an at-risk mental state for psychosis: a meta-analytic review. Front Psychiatry.

[64] Schultze-Lutter F, Klosterkotter J, Michel C, Winkler K, Ruhrmann S (2012). Personality disorders and accentuations in at-risk persons with and without conversion to first-episode psychosis. Early Inter Psychiatry.

[65] Pieters LE, Nadesalingam N, Walther S, van Harten PN (2022). A systematic review of the prognostic value of motor abnormalities on clinical outcome in psychosis. Neurosci Biobehav Rev.

[66] Callaway DA, Perkins DO, Woods SW, Liu L, Addington J (2014). Movement abnormalities predict transitioning to psychosis in individuals at clinical high risk for psychosis. Schizophr Res.

[67] Raballo A, Poletti M, Preti A (2020). Meta-analyzing the prevalence and prognostic effect of antipsychotic exposure in clinical high-risk (CHR): when things are not what they seem. Psychol Med.

[68] Schnack HG, Kahn RS (2016). Detecting neuroimaging biomarkers for psychiatric disorders: sample size matters. Front Psychiatry.

[69] Cannon TD, Yu C, Addington J, Bearden CE, Cadenhead KS, Cornblatt BA (2016). An individualized risk calculator for research in prodromal psychosis. Am J Psychiatry.

[70] Carrion RE, Cornblatt BA, Burton CZ, Tso IF, Auther AM, Adelsheim S (2016). Personalized prediction of psychosis: external validation of the NAPLS-2 psychosis risk calculator with the EDIPPP project. Am J Psychiatry.

[71] Zhang T, Li H, Tang Y, Niznikiewicz MA, Shenton ME, Keshavan MS (2018). Validating the predictive accuracy of the NAPLS-2 psychosis risk calculator in a clinical high-risk sample from the SHARP (Shanghai At Risk for Psychosis) program. Am J Psychiatry.

[72] Satterthwaite TD, Connolly JJ, Ruparel K, Calkins ME, Jackson C, Elliott MA (2016). The Philadelphia Neurodevelopmental Cohort: a publicly available resource for the study of normal and abnormal brain development in youth. NeuroImage.

[73] Hauke DJ, Schmidt A, Studerus E, Andreou C, Riecher-Rossler A, Radua J (2021). Multimodal prognosis of negative symptom severity in individuals at increased risk of developing psychosis. Transl Psychiatry.

[74] Koutsouleris N, Kambeitz-Ilankovic L, Ruhrmann S, Rosen M, Ruef A, Dwyer DB (2018). Prediction models of functional outcomes for individuals in the clinical high-risk state for psychosis or with recent-onset depression: a multimodal, multisite machine learning analysis. JAMA Psychiatry.

[75] Reniers RL, Lin A, Yung AR, Koutsouleris N, Nelson B, Cropley VL (2017). Neuroanatomical predictors of functional outcome in individuals at ultra-high risk for psychosis. Schizophr Bull.

[76] Bach S, Binder A, Montavon G, Klauschen F, Muller KR, Samek W (2015). On pixel-wise explanations for non-linear classifier decisions by layer-wise relevance propagation. PLoS ONE.

[77] Cannon TD (2016). Brain biomarkers of vulnerability and progression to psychosis. Schizophr Bull.

[78] Worthington MA, Cannon TD (2021). Prediction and prevention in the clinical high-risk for psychosis paradigm: a review of the current status and recommendations for future directions of inquiry. Front Psychiatry.

