# Supplementary material for: Predictors of transition in patients with clinical high risk for psychosis: an umbrella review
Source: Transl Psychiatry. 2023 Aug 28;13:286. doi: 10.1038/s41398-023-02586-0 (PMC10462748; doi:10.1038/s41398-023-02586-0)
Supplement: Supplementary file 2 — Prisma Checklist [file 41398_2023_2586_MOESM2_ESM.docx]

**Supplemental material**

Andreou C, Eickhoff S, Heide M, de Bock R, Obleser J, Borgwardt S. Predictors of transition in patients with clinical high risk for psychosis: An umbrella review

[Search strings 2](#_Toc104794743)

[Table S2: AMSTAR quality ratings of included papers 4](#_Toc104794744)

# Search strings

**Pubmed**

("Review"[Publication Type] OR "systematic review"[Publication Type] OR "meta analysis"[Publication Type])

AND

("clinical high risk”[All Fields] OR “at risk mental state”[All Fields] OR "high risk"[All Fields])

AND

("psychosis"[All Fields] OR "psychotic disorder"[All Fields] OR "schizophrenia"[All Fields])

AND

("prediction"[All Fields] OR "biomarker"[All Fields] OR “associat*”[All Fields])

*Filters*: Time range: Last 10 years; Language: English

**PsychInfo**

("prediction" or "biomarker" or “associat*”).mp. [mp=title, abstract, heading word, table of contents, key concepts, original title, tests & measures, mesh word]

AND

("psychosis" or "psychotic disorder" or "schizophrenia").mp. [mp=title, abstract, heading word, table of contents, key concepts, original title, tests & measures, mesh word]

AND

("clinical high risk" or "at risk mental state" or "high risk").mp. [mp=title, abstract, heading word, table of contents, key concepts, original title, tests & measures, mesh word]

limit  to (peer reviewed journal and english language and yr="2012 - 2022”)

limit to ("0830 systematic review" or "1200 meta analysis" or "literature review”)

**Scopus**

DOCTYPE(re)

AND

TITLE-ABS-KEY("clinical high risk" OR "at risk mental state" OR "high risk")

AND

TITLE-ABS-KEY("psychosis" OR "psychotic disorder" OR "schizophrenia") AND TITLE-ABS-KEY("prediction" OR "biomarker" OR “associat*”)

AND

LANGUAGE(english)

AND

PUBYEAR AFT 2011

**Cochrane library**

("Review" OR "systematic review" OR "meta analysis"):ti,ab,kw

AND

("clinical high risk" OR "at risk mental state" OR "high risk"):ti,ab,kw

AND

("psychosis" OR "psychotic disorder" OR "schizophrenia"):ti,ab,kw AND ("prediction" OR "biomarker"):ti,ab,kw

*Filters*: Date between 01.03.2012 and 01.03.2022; language: English

# Table S1: AMSTAR quality ratings of included papers

Column numbers correspond to the respective AMSTAR items: 1. A priori design; 2. Duplicate data selection; 3. Comprehensive literature search; 4. Publication status considered in data selection; 5. List of studies provided; 6. Characteristics of studies provided; 7. Quality assessment; 8. Consideration of quality in analyses; 9. Synthesis methods; 10. Publication bias; 11. Declaration of conflict of interest.

| **Authors** | **Year** | **1** | **2** | **3** | **4** | **5** | **6** | **7** | **8** | **9** | **10** | **11** | **total score** | **total possible** | **total score**  **(% max)** |
| --- | --- | --- | --- | --- | --- | --- | --- | --- | --- | --- | --- | --- | --- | --- | --- |
| Addington et al. | 2014 | no | no | no | yes | no | yes | no | yes | n/a | n/a | no | 3 | 9 | 33% |
| Bodatsch et al. | 2013 | no | no | no | no | no | no | no | no | no | no | yes | 1 | 9 | 11% |
| Boldrini et al. | 2019 | yes | yes | yes | yes | yes | yes | yes | yes | yes | yes | yes | 11 | 11 | 100% |
| Bora et al. | 2014 | no | no | yes | yes | yes | yes | no | no | yes | yes | yes | 7 | 11 | 64% |
| Brew et al. | 2018 | no | yes | yes | yes | yes | no | yes | no | n/a | n/a | no | 6 | 9 | 67% |
| Catalan A, Salazar De Pablo G, Aymerich C, et al. | 2021 | yes | yes | yes | yes | yes | yes | yes | yes | yes | yes | yes | 11 | 11 | 100% |
| Catalan A, Salazar de Pablo G, Serrano SV et al. | 2021 | no | yes | yes | yes | yes | yes | yes | yes | yes | yes | yes | 10 | 11 | 91% |
| Chaumette et al. | 2016 | no | yes | yes | yes | no | yes | yes | no | yes | no | yes | 7 | 11 | 64% |
| De Herdt et al. | 2013 | no | yes | yes | yes | yes | yes | no | yes | yes | yes | yes | 9 | 11 | 82% |
| Erickson M et al. | 2016 | no | yes | yes | yes | yes | yes | yes | yes | yes | yes | yes | 10 | 11 | 91% |
| Farris et al. | 2020 | yes | yes | yes | yes | yes | yes | no | no | yes | no | yes | 8 | 11 | 73% |
| Fortea et al. | 2021 | no | yes | yes | yes | yes | yes | yes | yes | yes | yes | yes | 10 | 11 | 91% |
| Fusar-Poli et al. | 2015 | yes | yes | yes | yes | yes | yes | yes | yes | yes | yes | yes | 11 | 11 | 100% |
| Fusar-Poli et al. | 2012 | no | yes | yes | yes | yes | yes | yes | yes | yes | yes | yes | 10 | 11 | 91% |
| Gogos et al. | 2019 | no | yes | yes | yes | yes | yes | no | no | n/a | n/a | yes | 6 | 9 | 67% |
| Hinney et al. | 2020 | yes | yes | yes | yes | yes | yes | no | yes | yes | yes | yes | 10 | 11 | 91% |
| Izon et al. | 2018 | yes | yes | yes | yes | yes | yes | yes | yes | n/a | n/a | yes | 9 | 9 | 100% |
| Karanikas and Garyfallos | 2015 | no | no | no | yes | no | yes | no | no | n/a | n/a | yes | 3 | 9 | 33% |
| Khoury and Nasrallah | 2018 | no | no | yes | yes | no | yes | no | yes | n/a | n/a | yes | 5 | 9 | 56% |
| Kraan et al. | 2016 | no | yes | yes | yes | yes | yes | yes | yes | yes | yes | yes | 10 | 11 | 91% |
| Malda et al. | 2019 | yes | yes | yes | yes | yes | yes | yes | yes | yes | yes | yes | 11 | 11 | 100% |
| Misiak et al. | 2021 | yes | yes | yes | yes | yes | yes | yes | yes | yes | yes | yes | 11 | 11 | 100% |
| Montemagni et al. | 2020 | no | yes | no | yes | yes | yes | no | yes | n/a | n/a | yes | 6 | 9 | 67% |
| Moore et al. | 2021 | yes | yes | yes | yes | yes | yes | yes | yes | n/a | n/a | yes | 9 | 9 | 100% |
| O’Donoghue et al. | 2016 | no | yes | yes | yes | yes | yes | yes | no | n/a | n/a | yes | 7 | 9 | 78% |
| Oliver et al. | 2020 | yes | yes | yes | yes | yes | yes | yes | yes | yes | yes | yes | 11 | 11 | 100% |
| Park and Miller | 2020 | no | no | yes | yes | yes | yes | no | no | yes | yes | yes | 7 | 11 | 64% |
| Peh et al. | 2019 | yes | yes | yes | yes | yes | yes | yes | yes | yes | yes | yes | 11 | 11 | 100% |
| Perrottelli et al. | 2021 | no | yes | yes | yes | yes | yes | no | no | n/a | n/a | yes | 6 | 9 | 67% |
| Pieters et al. | 2022 | yes | yes | yes | yes | yes | yes | yes | yes | n/a | n/a | yes | 9 | 9 | 100% |
| Raballo et al. | 2020 | no | yes | yes | yes | yes | yes | yes | yes | yes | yes | yes | 10 | 11 | 91% |
| Riecher-Rössler and Studerus | 2017 | no | no | no | yes | yes | yes | yes | yes | n/a | n/a | yes | 4 | 9 | 44% |
| Romeo et al. | 2020 | yes | no | yes | yes | no | yes | no | no | yes | yes | yes | 7 | 11 | 64% |
| Rosen et al. | 2021 | yes | yes | yes | yes | yes | yes | yes | no | n/a | n/a | yes | 8 | 9 | 89% |
| Schiavone and Trabace | 2017 | no | no | no | yes | yes | no | no | no | n/a | n/a | yes | 3 | 9 | 33% |
| Seabury and Cannon | 2020 | no | no | yes | yes | no | yes | no | yes | n/a | n/a | yes | 5 | 9 | 56% |
| Tor J et al. | 2018 | no | yes | yes | yes | yes | yes | no | yes | n/a | n/a | yes | 7 | 9 | 78% |
| Treen et al. | 2016 | no | no | yes | no | yes | yes | no | no | n/a | n/a | yes | 4 | 9 | 44% |
| van der Steur et al. | 2020 | no | yes | yes | yes | yes | yes | no | yes | n/a | n/a | no | 6 | 9 | 67% |
| van Donkersgoed et al. | 2015 | no | yes | yes | yes | yes | yes | yes | yes | yes | yes | yes | 9 | 11 | 82% |
